# Spatial Reorganization of Myoelectric Activities in Extensor Digitorum for Sustained Finger Force Production

**DOI:** 10.3390/s19030555

**Published:** 2019-01-29

**Authors:** Zhixian Gao, Shangjie Tang, Xiaoying Wu, Qiang Fu, Xingyu Fan, Yun Zhao, Lintao Hu, Lin Chen, Wensheng Hou

**Affiliations:** 1 Key Laboratory of Biorheological Science and Technology, Ministry of Education, Bioengineering College, Chongqing University, Chongqing 400044, China; 20141901009@cqu.edu.cn (Z.G.); 20161902051@cqu.edu.cn (S.T.); x.y.wu@cqu.edu.cn (X.W.); 20161901009@cqu.edu.cn (Y.Z.); 20171901037@cqu.edu.cn (L.H.); w.s.hou@cqu.edu.cn (W.H.); 2 Chongqing Engineering Research Center of Medical Electronics Technology, Chongqing 400044, China; xingyu.fan02@gmail.com; 3 Chongqing Key Laboratory of Artificial Intelligence and Service Robot Control Technology, Chongqing 400044, China; 4 College of Automation, Harbin Engineering University, Harbin 150001, China; frannnnnk@hrbeu.edu.cn

**Keywords:** spatial reorganization, inhomogeneous muscle activity, multi-channel surface electromyography, neuromuscular compartment, sustained contraction

## Abstract

The study aims to explore the spatial distribution of multi-tendinous muscle modulated by central nervous system (CNS) during sustained contraction. Nine subjects were recruited to trace constant target forces with right index finger extension. Surface electromyography (sEMG) of extensor digitorum (ED) were recorded with a 32-channel electrode array. Nine successive topographic maps (TM) were obtained. Pixel wise analysis was utilized to extract subtracted topographic maps (STM), which exhibited inhomogeneous distribution. STMs were characterized into hot, warm, and cool regions corresponding to higher, moderate, and lower change ranges, respectively. The relative normalized area (normalized to the first phase) of these regions demonstrated different changing trends as rising, plateauing, and falling over time, respectively. Moreover, the duration of these trends were found to be affected by force level. The rising/falling periods were longer at lower force levels, while the plateau can be achieved from the initial phase for higher force output (45% maximal voluntary contraction). The results suggested muscle activity reorganization in ED plays a role to maintain sustained contraction. Furthermore, the decreased dynamical regulation ability to spatial reorganization may be prone to induce fatigue. This finding implied that spatial reorganization of muscle activity as a regulation mechanism contribute to maintain constant force production.

## 1. Introduction

The function of human daily movements are implemented through skeletal muscle contraction activating motor units (MU) under central nervous system (CNS) modulation [1]. Generally, a motor unit consists of a motor neuron and skeletal muscle fibers with specific distribution that innervated by that neuron. During motor behaviors, the MU recruitment patterns can be affected by various factors associated with motor function and muscular status such as pain, fatigue, force variability, etc. [2,3,4]. Thus, MU recruitment strategy changes will consequently cause fluctuations in the spatial distribution of muscle activity [5,6]. In order to maintain constant force output, the CNS needs to modulate the MU recruitment strategies of skeletal muscle dynamically, which will directly impact on corresponding muscle activity distribution. Therefore, analyzing the spatial distribution variations of muscle activity during sustained contraction may provide an insight into control strategies of the neuromuscular system. Pilot studies have employed spatial distribution of muscle activity to multiple biomedical applications, such as isometric muscle force estimation and muscle-tendon units localization [7,8]. Furthermore, the activity distribution analysis tended to provide a noteworthy robust improvement for identification of fine actions such as thumb rotation and multiple DoF (degree of freedom) control in surface electromyography (sEMG)-based robotics and assistive technologies [9,10]. Changes in spatial distribution of myoelectric activity have been observed in multiple muscles, which are related to the regulation mechanism of muscular activity. According to previous studies, Farina et al. measured sEMG signals in upper trapezius muscle and reported that the changes in spatial distribution of muscle activity played an important role to sustained contractions [5]. Furthermore, Christian et al. studied biceps brachii muscle via isometric elbow flexion contraction tasks, and also found the spatial reorganization of muscle activity correlated to the modulation of tangential force [6]. Moreover, the spatial reorganization of muscles may reflect the muscular status. Castroflorio et al. manifested that a unilateral painful simulation could induce spatial reorganization in masseter muscle [11].

In order to detail the functional components of motor behaviors, various medical imaging techniques have already been involved to map the spatial muscle activity information. For example, Mariappan et al. [12] localized the regional neuromuscular compartments with magnetic resonance imaging, and found different functional distributions in flexor digitorum profundus (FDP) and flexor digitorum superficialis (FDS) according to vibration tasks performed by different individual fingers. Sofia et al. [13] dynamically measured the muscular features (such as muscle cross-sectional area, pennation angle, contraction patterns, etc.) using ultrasound and identified the muscle architectures of forearm extensor correlated significantly to the finger extension force production. However, Magnetic Resonance Imaging is not suitable for dynamic neuromuscular activation measurements during continuous force production, while ultrasound imaging is only suitable for detecting low levels of muscle activity [14]. Electrode-array-based multi-channel surface electromyography (sEMG) is a new technology capable of simultaneously sampling multi-point myoelectric activity of skeletal muscle [15], which potentially provides detailed information for spatial organization of muscle activity [16].

With the multi-channel sEMG signals, a few studies have already illustrated the relationship between activity distribution and the force level and/or direction of muscle contraction [7,8,17]. According to recent research, the gravity center of sEMG topographic maps (TM) was usually considered as an acceptable measurement to investigate the distribution and inhomogeneity of activity level in a skeleton muscle [2,5]. However, the activity of neuromuscular compartment distributed dynamically across different spatial areas over time during prolonged muscle contraction, whereas the conventional used gravity center parameter only represents the center distribution according to static measurements. In addition, the gravity center neglects the magnitude changes in different regions of muscle activity. Our previous results observed that shifts for the center of gravity were limited along both the fiber direction and the perpendicular direction (less than 1 mm) in multi-tendoned forearm muscles according to multi-finger muscle activity, while the spatial distribution of TM illustrated visible regional magnitude changes with force levels [18]. This suggested that the gravity center may not fully describe the changes of TM for different motor tasks over endurance time, which deteriorate evaluating the spatial distribution of muscle activities.

According to physiological theory, multi-tendinous muscles in forearm plays key role serving the dexterous movement of fingers [19]. The presented work aimed to study the regulation mechanism of spatial distribution in multi-tendinous muscles during sustained muscle contractions. The changes in spatial distribution of sEMG activity in forearm ED according to sustained constant index finger extension were investigated based on electrode-array-based sEMG technology. We analyzed the spatial distribution change via TM subtraction over time as a surrogate to the neuromuscular control strategy during sustained contraction. The results suggested that reorganization of spatial distribution can be captured based on TM subtraction, which may provide an innovate insight to understand the regulation mechanism of reorganization during sustained contraction.

## 2. Methods

### 2.1. Subjects

The protocol of this study was approved by the local ethics committee. Nine right-handed male subjects (age 24 ± 1.2 years; height 171.2 ± 3.7 cm; and weight 66.4 ± 7.1 kg) were recruited. All subjects were confirmed free of any formal neurological, musculoskeletal, or orthopaedic disorders, and no intensive exercise before the experiment. Informed consent was obtained before the subject participated.

### 2.2. Experimental Design

**Figure 1:**
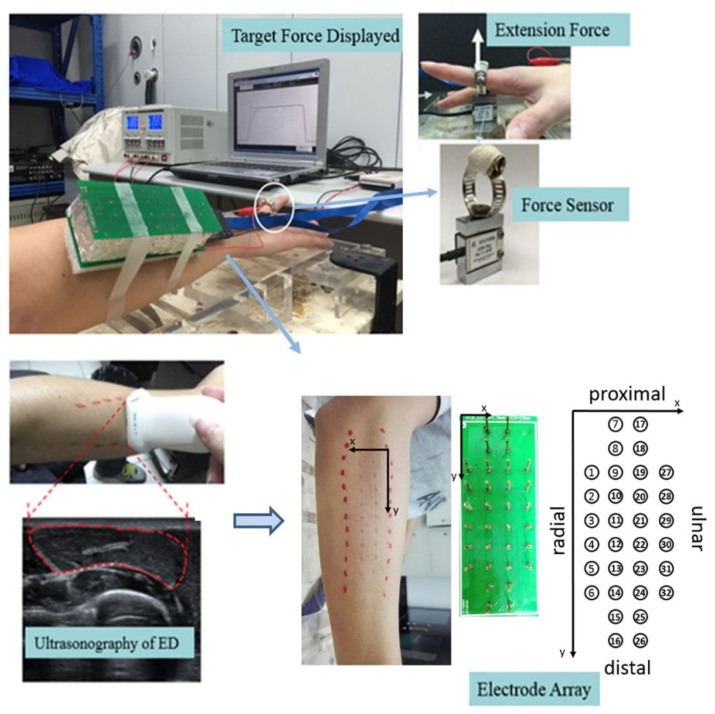
The scene of the experiment for sustained force-tracking through right index finger extension. The extension force of index was detected with a custom-made ring-like force sensor, and the sEMG was recorded with 32-channel circular electrodes of test probes. The edge of superficial region of ED muscle was identified using ultrasonography.

Subjects sat upright in neutral position, with right forearm placed in pronation and the elbow joint flexed at around 120°. Straps were used to avoid unexpected movements. The proximal phalanx of right index finger was required to put into the ring of a customized force transducer (JLBS-5kg, Bengbu Sensor Inc., Bengbu, China) to record the finger extension force (Figure 1).

Subjects were instructed to extend the right index fingers following visible target force cue. The index finger extension strength was required to match a pre-specified target force level, whereas other fingers (middle, ring, and little finger) kept relax. Maximal voluntary contractions (MVC) were measured for every subject before taking participation. Three 3 s maximal-forced contractions with 5 min interval were performed, and the max strength was defined as MVC. Then, after a 10 min rest, subjects were asked to conduct three force-tracking tasks, at 15%, 30%, and 45% MVC, respectively. The target force and actual force production of index finger were displayed on the screen in front of the subject (Figure 1). Subjects were required to maintain the extension force matched with target force for 90 s in tasks. Every force-tracking task was repeated three times. The trials of submaximal contraction were performed with an interval of 5–10 min. A training session was assigned for subjects to get familiar with the experimental procedure before the test.

### 2.3. Signal Acquisition

The edge of superficial region of ED muscle was identified using ultrasonography (DC-8, Mindray, Shenzhen, China). The ultrasonic linear probe was placed on top of ED skin and moved across the muscle fibers. A red marker was made when ultrasound detected the edge of ED muscle (Figure 1). The ultrasonography scanning was performed in the second affiliated hospital of Chongqing medical university.

The sEMG signals were collected from the ED muscle with 32 circular electrodes of test probes (WAROM Group, Shenzhen, China). The probes were configured as a matrix of 6, 10, 10, and 6 electrodes in 4 adjacent columns, respectively, with a 10 mm inter-electrode distance (Figure 1). Prior to the electrode array placement, the skin was shaved, abraded and cleaned with alcohol. Myoelectric signals were recorded using Cerebus (BlackRock MicroSystem, Salt Lake City, UT, USA) with a head stage follower plugged in the socket of the electrode array. Both the reference and ground electrodes were placed on the right wrist. The 32-channel sEMG signals and force data were synchronously sampled at 2000 Hz, and sEMG signals were amplified (-3dB bandwidth 10–500Hz) by a gain of 300 times.

### 2.4. Data Processing

**Figure 2:**
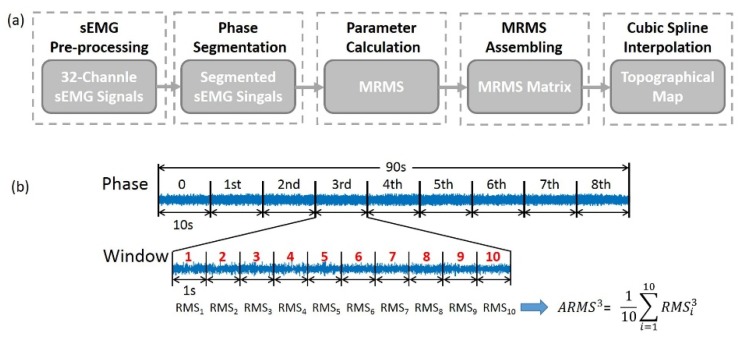
(**a**) Block diagram of topographical maps generation. (**b**) Example of averaged RMS calculated for the 3rd phase.

As shown in Figure 2a, sEMG topographic maps (TM) were generated to present the spatial distribution of muscle activities in ED during force-tracking tasks. For pre-processing, sEMG signals were filtered using MATLAB (Version 2015, the Mathworks Inc., Natick, MA, USA) through a band-pass filter (four order Butterworth, 20–500 Hz) and an adaptive filter in order to remove the power line interference, noise, etc. To investigate the changes of myoelectric activities during sustained submaximal contraction, the recorded 90 s sEMG signals per trail were segmented into 9 phases (10 s per phase, respectively).

#### 2.4.1. Visualization of 2D Myoelectric Activities of ED

To evaluate the intensity of myoelectric activities, the mean RMS (MRMS) was calculated phase by phase for every force level with the following steps (Figure 2b):

(1) RMS values [20] were sequentially calculated with a 1 s non-overlap time window through each phase. Therefore, a total of 10 RMS values were obtained for every 10 s phase.

(2) The averaged RMS (ARMS) values $ARMS^{n}$ for the *n*th phase (*n* = 0, 1, 2, …, 8) were calculated as

(1) $$ARMS^{n}=\frac{1}{10}\overset{10}{\underset{i=1}{\sum }}RMS_{i}^{n}\;\;\left(i=1,2,\ldots ,\;10\right)$$

and

(3) the final mean RMS (MRMS) values of the *n*th phase were obtained by averaging $ARMS^{n}$ from all the 3 repeated trails. The above analysis was performed channel by channel through all 32 channels.

**Figure 3:**
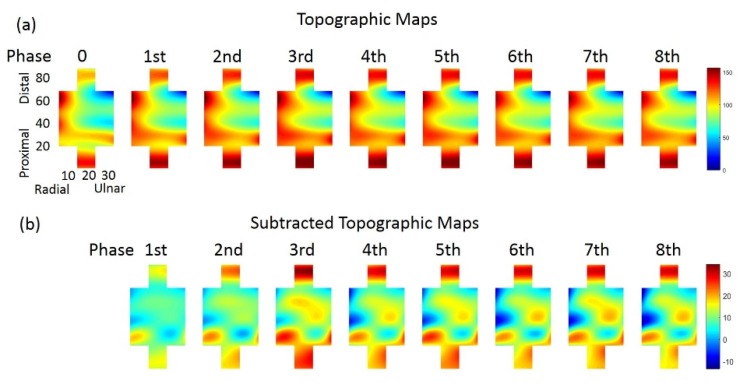
(**a**) Nine continuous TMs at 45%MVC of one subjects. (**b**) Eight continuous STMs extracted from the 9 continuous TMs at 45%MVC of the same subject in (**a**).

To describe myoelectric activities distribution in ED, MRMS matrix (4 × 10 grid) was assembled according to the electrode array configuration, with elements that corresponds to no electrodes were set as ‘NAN’. Multi-directionally interpolation method were utilized with cubic spline functions (factor is 10) to transfer the MRMS matrix into TM (31 × 91 grid). Nine successive 2D TMs were extracted to represent corresponding myoelectric activities in 9 phases during one sustained contraction (Figure 3a).

#### 2.4.2. Quantification of the Myoelectric Activities in sEMG TM

As the pixel value of TM represented the intensity of myoelectric activities at the corresponding point of muscle, the total intensity of myoelectric activities for a topographic map can be estimated as the following formula

(2) $$I^{n}=\sum M^{n}\left(i,j\right)\;\left(n=0,1,2,\cdots ,8\right)$$

where $I^{n}$ represents the total intensity of the TM at *n*th time phase, $M^{n}\left(i,j\right)$ represent the i×jth element (i = 1,2,…,31; j = 1, 2, …,91) in the TM (31 × 91 grid) at the *n*th phase. Thus, a sequence consists of 9 $I^{n}$ could be obtained to characterize the total intensity of myoelectric activities over time. To learn the changing trend of $I^{n}$ that corresponds to a certain force level, the 9-$I^{n}$ sequence was normalized by its initial time phase $I^{0}$.

#### 2.4.3. Changes of Myoelectric Activities Distribution in ED

A pixel subtraction method was used to quantify the changes of myoelectric activities distribution in ED based on originally obtained TMs (Figure 3a). Pixel-to-pixel subtractions were performed as subtracting the initial TM (M^0^) from TMs over time (M^1^–M^8^), therefore, 8 corresponding subtracted TMs (STMs, symbolized as dM^1^-dM^8^ below, Figure 3b) were obtained as following

(3) $$dM^{n}\left(i,j\right)=M^{n}\left(i,j\right)-M^{0}\left(i,j\right)\;\left(i=1,2,\ldots ,31;\;j=1,2,\ldots ,91\right)$$

where $M^{0}$ referred to TM at the initial phase (n = 0), $M^{n}$ was the sequence of TM at the rest eight time phase, and $dM^{n}$ represented STM at the *n*th time phase (n=1,2,⋯,8) during a constant contraction task.

**Figure 4:**
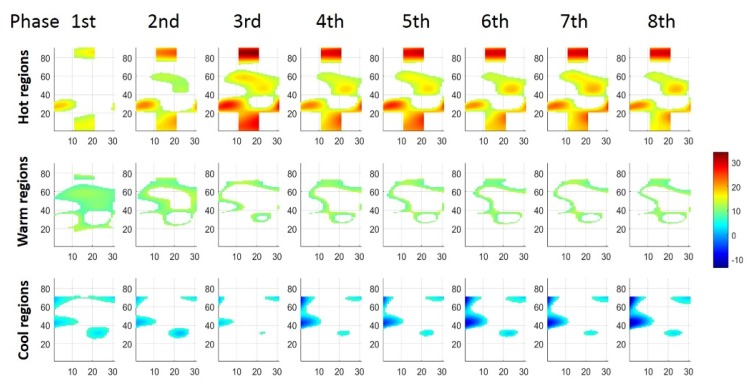
Hot regions, warm regions and cool regions at 8 phases during sustained index extension (same subject as Figure 3).

As shown in Figure 3b, STMs visually exhibited inhomogeneous changes of myoelectric activities distribution in ED. To analyze the fluctuating regions during tasks, we characterized STM into three regions according to the following criteria:

(4) $$\mathrm{Hot}\;\mathrm{regions}:\;dM\left(i,j\right)\geq Q_{3}\;\mathrm{Warm}\;\mathrm{regions}:\;Q_{1}<dM\left(i,j\right)<Q_{3}\;\mathrm{Cool}\;\mathrm{regions}:\;dM\left(i,j\right)\leq Q_{1}$$

where dM(i,j) represented the i×jth element in the STM ($dM^{n}$), which was the differences between original topographic maps ($M^{n}$ to $M^{0}$, n = 1,2,…,8). $Q_{1}$ and $Q_{3}$ were the first and third quartile according to all the dM values in $STM^{1}$ of the first phase ($dM^{1}$). We named the regions within the interquartile range (IQR) of all dM values in $dM^{1}$ as warm regions, the regions with larger changes ($\geq Q_{3}$) as hot regions, and the regions with smaller changes ($\leq Q_{1}$) as cool regions (Figure 4).

To analyze the change trend of three different regions in STMs, corresponding region areas were computed through pixel number counts. Then, all region areas (hot, warm, cool regions, respectively) of STMs ($dM^{n}$) was normalized to the corresponding areas within the STM of the first phase ($dM^{1}$). We defined the relative normalized regional area as follows:

(5) $$AR^{n}=\frac{A^{n}}{A^{1}}$$

where $A^{n}$ is the region area at the *n*th time phase (n=1,2,⋯,8), and the region area at the 1st time phase $A^{1}$ was taken as the reference area. The parameter $AR^{n}$ employed the ratio of $A^{n}$ and $A^{1}$ to represent the relative regional area of $dM^{n}$ at the *n*th time phase that normalized to the $dM^{1}$.

### 2.5. Statistical Analysis

Two-way analysis of variance (ANOVA) was performed based on the relative normalized regional areas according to different force levels and time phases (3 force levels × 8 time phases). Mauchly Test was used to characterize sphericity, and a Greenhouse-Geisser correction was used to modify the degree of freedom when sphericity was significant. Post hoc pairwise multiple comparisons with the Tukey HSD correction method were used when necessary. Similarly, Two-way ANOVA was also used to evaluate averaged relative normalized regional area of STMs among different force levels and regions (3 force levels × 3 regions). In addition, one-way ANOVA was applied on the averaged intensity of TM and the slope of normalized intensity among force levels. The Statistical analysis was carried out using SPSS 21 (SPSS Inc., Chicago, IL, USA). Significance was accepted for *p*-values less than 0.05.

## 3. Results

### 3.1. Performance of Force Output and Changes of TM during Sustained Contraction

**Figure 5:**
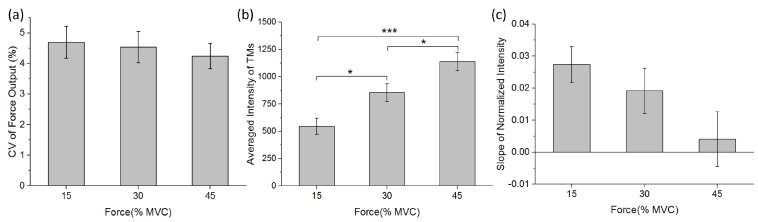
(**a**) The coefficient of variation (CV) of force output at three force levels during sustained constant index finger extension; (**b**) the averaged intensity of 9 TMs at three force levels; and (**c**) the slope of normalized intensity for 9 continuous TMs at three force levels. Asterisks indicate significant difference in multiple compares (* 0.01 < *p* < 0.05; *** *p* < 0.005).

All nine subjects conducted the force-tracking tasks under instructions. The force productions during task performance were measured as 15.01 ± 0.69, 29.97 ± 1.31%, and 44.94 ± 1.93% MVC, which well-matched the target force level of 15%, 30%, and 45% MVC, respectively. Variations of force output were calculated, which was less than 5% for all force levels, as illustrated by Figure 5a. The results indicated that all subjects maintained stable force output with index finger extension according to task requirements.

Figure 3a showed an example of right ED TM sequenced in nine time phases according to the sustained index finger extension at 45% MVC for one subject. The sequence of topographic maps visually illustrated a dynamic modulation procedure upon the intensity of myoelectric activities over time. The intensity distribution and inhomogeneity fluctuated to satisfy the task-related force maintain requirements. It was also clear that higher level of muscle activation on TM primarily corresponds to the radial-proximal regions of ED, and the intensity of which increased as well as region areas gradually enlarged during the sustained contraction task. In other words, different parts of ED contributed differently to index finger extension, and the contributions were modulated to maintain the constant force output.

Figure 5b,c reported the averaged intensity values among 9 TMs and the linear regression slopes of normalized intensity (as $I^{n}$ normalized to the initial time phase $I^{0}$) against 9 time phases in ED at three force levels, respectively. One-way ANOVA indicated that force level significantly correlated to the activity intensity of the ED (*F*(2,26) = 14.838, *p* < 0.001). Tukey HSD test revealed that the activity intensity increased significantly as force levels raised (15% MVC vs. 30% MVC, *p* = 0.024; 15% MVC vs. 45% MVC, *p* < 0.001; and 30% MVC vs. 45% MVC, *p* = 0.039). The slopes of normalized intensity tend to decrease as force increase; the relationship was not significant however.

### 3.2. Distribution Variation of STMs at Three Force Levels during Sustained Contraction

**Figure 6:**
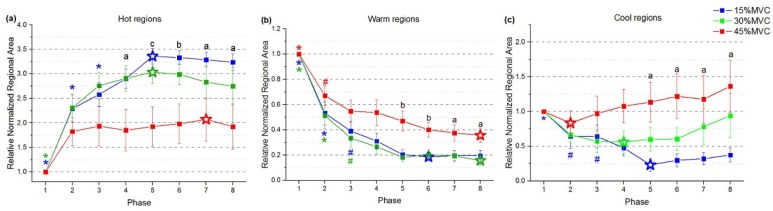
The relative normalized regional area of hot regions (**a**), warm regions (**b**), and cool regions (**c**) during sustain constant index finger contractions at three force levels. ‘☆’ represent the extreme values. Asterisk and octothorpe indicate significant difference compared with the extreme values (* *p* < 0.005; # *p* < 0.05). Different lowercase letters indicate significant effect among force levels (a 0.01 < *p* < 0.05; b 0.005 < *p* < 0.01; c *p* < 0.005).

The relative normalized regional area of STMs at different force level (Figure 6) was analyzed. The relative normalized regional area tended to have specific changing trends corresponds to hot, warm and cool regions respectively during sustained constant index finger extension. The changing trend was ascending within the hot regions, but descending in warm regions. For cool regions, however, the relative area changes turned to be descending at 15% MVC and ascending at 45% MVC. Furthermore, the changing trends of three regions were affected by force. We defined parameters as rising time, plateauing time and falling time to investigate the changes within specific regions. Therefore, the plateauing time was defined as the period that the relative normalized regional area values reached and maintained a relatively stable maximum/minimum level; while the rising/falling time was the early dynamic period that the relative normalized regional area values increasing/decreasing from the first phase to the plateauing time. The extreme value (maximum/minimum, ‘☆’ in Figure 6) of averaged relative regional area were used to characterize rising, plateauing, and falling time, respectively. When compared to the extreme value, relative normalized area within a continuous time phases with no significant difference according to post hoc tests were characterized as ‘plateauing time’, while relative area values within the phases exhibited significant difference to extreme were characterized as ‘rising time’/‘falling time’ in increased/decreased curves.

For hot regions, the relative normalized regional area at three different force levels required different rising time. According to Figure 6a, the statistical results indicated that the rising time lasted from phase 1 to 3 at the force of 15% MVC (blue), but occupied the first phase only at 30% MVC (green). When performing tasks with the force level of 45% MVC, the plateau started within the first phase (red). For warm regions (Figure 6b), the relative regional areas exhibited a decreased trend at all force levels, as the ‘falling time’ of 15% (blue) and 30% MVC (green) lasted from phase 1 to 3. Contraction with 45% MVC (red) clarified a shorter falling time included the first two phases. For cool regions (Figure 6c), the trend of the relative normalized area change showed distinct differences according to force levels. At 15% MVC (blue), the relative area declined during the first three time phases and then reached the plateau, while plateauing time at 30% (green) and 45% MVC (red) lasted across all time phases. These results indicated that, the relative normalized regional areas according to the three regions (hot, warm, and cool) took a longer variation course (rising/falling time) at lower force level (15% and 30% MVC), whereas they kept relatively stable at 45% MVC from the beginning of contraction.

### 3.3. Regionalization Distribution of STMs at Three Force Levels during Sustained Contraction

**Figure 7:**
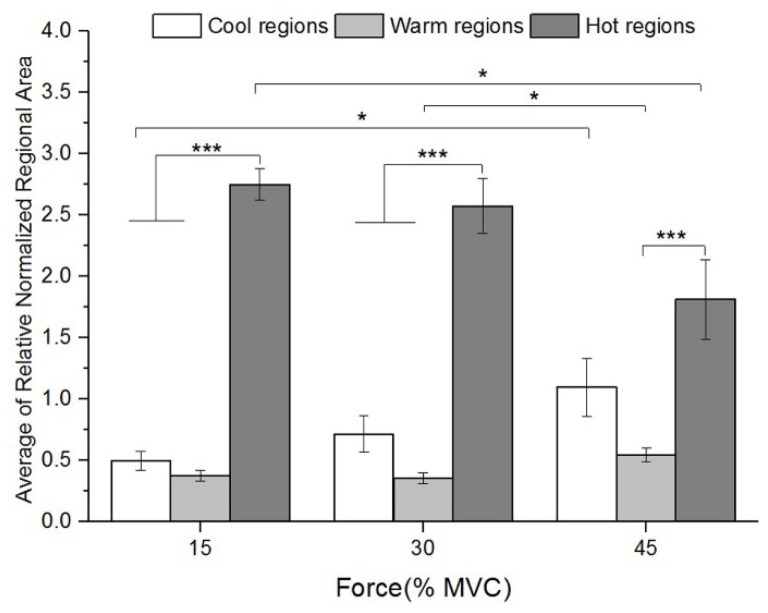
The average of relative normalized regional area of 8 STMs at three force levels. Asterisks indicate significant difference in multiple compares (* 0.01 < *p* < 0.05; *** *p* < 0.005).

Results also showed the relative normalized regional areas may be correlated to force level influence (Figure 6). For hot regions, the normalized relative area were higher at 15% and 30% MVC than at 45% MVC from phase 4 to 8. Values of relative normalized areas were found to be similar at 15% and 30% MVC within warm regions, while the corresponding values were much higher at 45% MVC from phase 5 to 8. For cool regions, highest relative normalized area values were observed at 45% MVC, and the last 4 phases while maintaining 30% MVC. These results revealed that the area of three regions (hot, warm, and cool) were regulated at different force levels. According to Figure 7, comparing with the lower force levels, the normalized relative area were lower in hot regions (15% vs. 45% MVC, *p* = 0.028), and higher in warm and cool regions at 45% MVC (warm: 15% vs. 45% MVC, *p* = 0.050; 30% vs. 45% MVC, *p* = 0.025; and cool: 15% vs. 45% MVC, *p* = 0.046). Therefore, the regulation of averaged relative normalized regional area among three regions were correlated to force level change.

## 4. Discussions

In this study, we used multi-channel sEMG technology to detect the electromyography activity of forearm ED according to the right index finger under sustained contractions with constant force level. Parameters of topographical sEMG maps were calculated to investigate the myoelectric activity intensity distribution of extensor digitorum. Our results suggested the sEMG activity had a non-uniform distribution according to sustained contractions of the index finger. The STMs of ED were divided into three regions with higher (hot), moderate (warm), and lower (cool) change. The changing trend of relative normalized regional area over endurance time was ascending within hot regions and descending within warm regions, while the situation varied for cool regions. Therefore, different regions of ED may contribute with different extents to maintain constant force production.

### 4.1. Regionalization of ED Activity

Skeletal muscle contraction is implemented by recruiting the MUs distributed in different muscle regions, and the regionalized muscle activities could be expected when local MUs were recruited. In the present study, we found that the activity intensity of ED increased not only corresponded to higher force level, but also over the endurance time as the positive slopes of normalized intensity indicated. However, the parameters based on averaged intensity according to RMS in TMs of all channels were global parameters that ignored the spatial distribution. On the contrary, TMs includes the information from multiple channels assembled in an electrical array, can map the spatial distribution of muscle activity intensity in detail. In our study, TMs illustrated the activated intensity had inhomogeneous distributions, and the higher intensity mainly concentrated in radial-proximal of ED. Moreover, compared to the initial epoch (M^0^), changes of myoelectric activity in ED (STM) also presented inhomogeneous distribution. According to the distribution change range (IQR according to dM^1^), STMs generally consist of three regions (hot, warm and cool). The results of analysis revealed that each region occupied a certain area in accordance with the non-uniform muscle activity changes in ED. This non-uniform changes may correlate to the unevenly distributed MUs, through which the recruitment strategy [3,21,22] and the firing rate [23,24] were regulated by CNS. In addition, the ED is a multi-tendoned muscle with neuromuscular compartment (NMC), and previous studies have found NMC in forearm multi-tendinous muscle [19] plays a crucial role for independent and dexterous finger motions. Selectively activated NMCs have been detected for different finger tasks by medical imaging techniques [12,13]. Inhomogeneous distribution of sEMG activity has also been found in multi-tendinous muscle due to selectively activated NMCs [16]. Since our experiment tasks were designed as index finger extension, selectively activated NMCs in ED should also result in the inhomogeneous distribution.

### 4.2. Reorganization in ED to Maintain Sustained Constant Force Output of Index Finger

During sustained constant contraction, CNS regulates the muscle activity from multiple temporal and spatial dimensions. Previous research have evaluated the spatial distribution of muscle activity, results suggested that the gravity center of topographic sEMG maps shifted over time and the myoelectric activity exhibited reorganization in sustained contraction conditions [5,6,25]. Our results suggested that it was the hot regions within STMs contributes primarily at low force levels (15% and 30% MVC); while the warm and cool regions steadily increased their contributions at higher force level (45% MVC). The fluctuations between hot and cool regions over time at different force levels indicated the reorganization of muscle activity in ED during sustained index finger contractions may correlate to the regulation strategy that facilitate maintaining a constant force. It has been reported that in order to maintain a certain force output, the spatial muscle activity distribution would be regulated under the peripheral and central mechanisms [5,6]. Cristian et al. [6] observed the spatial reorganization at the intensity of biceps brachii muscle activity in elbow flexion contractions, and clarified the center of topographical sEMG maps shifted with the direction of tangential force. The author attributed spatial reorganization to the changes in EMG amplitude [1] induced by the fluctuation of conduction velocity [26,27] and the intracellular action potential [28,29], or the central factors of MU discharge rates [23,24] and MU recruitment/de-recruitment.

On the other hand, to maintain constant force, CNS control muscle contractions by regulating MU recruitment patterns, such as the number and type of recruited MU and their firing rate [21]. This regulation can be represented indirectly by relative sEMG activity changes. In our study, the relative normalized regional areas of three regions were also affected by the factor of endurance time, which indicated that the myoelectric reorganization changing over time. At low force levels, the relative normalized regional areas of hot and cool regions reached a stable status much slower than at higher force levels. The longer dynamic period indicated a stronger regulation ability related to low force levels during sustained constant contraction. This can be explained as when contraction performed at low force level, CNS can improve modulation since MU recruitments were mainly related to slow, low-threshold MUs that generally corresponded to slow fatigue [30,31]. Furthermore, the alternative MU recruitment during sustained contraction at lower force levels may also offer support for the dynamic spatial reorganization in ED. However, it was reported that this alternative recruitment of MUs only occurred at relatively low-level muscle contraction [21]. Hence, by contrast, at higher force level, the disappeared dynamic period suggested declined dynamical regulation ability for spatial myoelectric distribution. In order to produce high force output, the fast, high-threshold MUs are activated. This type of MUs are quickly fatigued, and were easily de-recruited when they got fatigued [30,31]. Moreover, Kukulka et al. found that most of MUs were recruited at approximately 50% MVC in the adductor pollicis, which left almost no candidate MUs for additional recruitments and deteriorate the neuromuscular regulation insufficiency [32]. Additionally, Kohei Watanabe et al. reported the selectivity of muscle activations in rectus femoris enhanced with increasing force production [2]. Our study also agreed that the stability of spatial distribution in ED strengthened with force level during sustained constant contraction. However, the muscle region with stable relative area, but reduced modulation ability at higher force level, generally indicated a faster induction of fatigue.

## 5. Conclusions

The present study quantified the sEMG activation strategy in ED during sustained force production of index finger. The spatial distribution and recruitment characteristics of muscle activity were investigated based on STMs acquired via sEMG electrode array. The pixel wise analysis of subtracted topographical maps indicated three primary regions of sEMG activity were modulated in different modes according to different force levels, which was in accordance with the regionalization of ED. Furthermore, the reorganization of sEMG activity would decline over time, and this regulation ability loses can be aggravated at higher force level. The study suggested a potential way for spatial myoelectric activity distribution analysis, which may assist a better understanding of the regulation strategies of CNS during sustained constant contraction. However, the presented study designed sustained contraction tasks according to index finger extension only. In future, the work should be extended with other fingers. In addition, it would be necessary to involve electrode array with higher density for a better resolution of sEMG signal acquisition, in order to obtain more accurate STM.
